# Subsystem domination influence on magnetization reversal in designed magnetic patterns in ferrimagnetic Tb/Co multilayers

**DOI:** 10.1038/s41598-020-80004-x

**Published:** 2021-01-13

**Authors:** Łukasz Frąckowiak, Feliks Stobiecki, Gabriel David Chaves-O’Flynn, Maciej Urbaniak, Marek Schmidt, Michał Matczak, Andrzej Maziewski, Meike Reginka, Arno Ehresmann, Piotr Kuświk

**Affiliations:** 1 Institute of Molecular Physics, Polish Academy of Sciences, Poznań, Poland; 2 Faculty of Physics, University of Białystok, Białystok, Poland; 3 Institute of Physics and Center for Interdisciplinary Nanostructure Science and Technology (CINSaT), University of Kassel, Kassel, Germany

**Keywords:** Magnetic properties and materials, Surfaces, interfaces and thin films

## Abstract

Recent results showed that the ferrimagnetic compensation point and other characteristic features of Tb/Co ferrimagnetic multilayers can be tailored by He^+^ ion bombardment. With appropriate choices of the He^+^ ion dose, we prepared two types of lattices composed of squares with either Tb or Co domination. The magnetization reversal of the first lattice is similar to that seen in ferromagnetic heterostructures consisting of areas with different switching fields. However, in the second lattice, the creation of domains without accompanying domain walls is possible. These domain patterns are particularly stable because they simultaneously lower the demagnetizing energy and the energy associated with the presence of domain walls (exchange and anisotropy). For both lattices, studies of magnetization reversal show that this process takes place by the propagation of the domain walls. If they are not present at the onset, the reversal starts from the nucleation of reversed domains and it is followed by domain wall propagation. The magnetization reversal process does not depend significantly on the relative sign of the effective magnetization in areas separated by domain walls.

## Introduction

Tailored magnetic domain patterns and domain walls possess a multitude of applications. They are used in data storage devices^1–3^, in a variety of concept proofs for a controlled transport of magnetic particles^4–6^ in lab-on-chip devices, and in fundamental investigations^7,8^. For tailoring magnetic patterns in magnetic thin film systems three classes of methods are currently known: (1) thermal patterning^9^ by a tip of a scanning force microscope, (2) laser patterning^10–12^, and (3) patterning by light keV-ions^13,14^. In the present work we will focus on consequences of recent results, where it has been found that domains without domain walls (DWs) can be created in Tb/Co ultra-thin layer systems by bombardment with keV–He^+^-ions^15^. In the present context, by domains in ferrimagnetic films we understand regions with distinct effective magnetization orientation and by DW we denote the transition region between two such domains with a continuously rotating magnetization of both subsystems^15–17^. With these definitions in mind, it is possible to conceive magnetic configurations realizable in ferrimagnetic heterostructures with regions of different subsystem domination. Examples of such structures abounded in the literature many years ago. With respect to the position of the interface between distinctly dominated regions these ferrimagnetic heterostructures can be divided in two groups: (i) the interface lies in the sample plane^16–18^, (ii) the interface is parallel to the surface normal^15,19–22^. In such systems, for magnetic fields high enough to parallelly orient the effective magnetization of the regions with different types, a DW is created on the interface. This specific DW is called compensation DW^19,21,22^, and in the case of exchange coupled double layers (ECDL), interfacial DW (IDW)^17,18^. For diffuse composition at the interface, and consequently smooth variation of compensation temperature, the position of the compensation DW is strongly temperature dependent^20–22^. However, for a quasi-discontinuous composition change, the compensation DW is well localized^19^ and insensitive to small temperature changes. For large temperature variations, the ferrimagnetic structure changes from behaving as coexisting regions with distinct subsystem domination to behaving as regions similarly dominated but with different coercive fields^23^. Therefore, in ferrimagnetic films similar changes in magnetic properties can be achieved controlling either composition or temperature^24^.

While there is a considerable number of published works on modifications of ferromagnetic or exchange-bias systems by light-ion bombardment (IB)^13,25–36^ similar investigations for ferrimagnetic systems are infrequent and have primarily been conducted on garnet films^37^.

In one preceding work^15^ about a ferrimagnetic Tb/Co system, composed of Tb/Co multilayers with sublayers thin enough (*t*_Co_, *t*_Tb_ ≤ 1.5 nm) to render the stacks indistinguishable from alloys^38–42^, IB has been shown to modify the two magnetic subsystems of Co and Tb differently: the bombardment changes the Tb magnetic subsystem much more than that of Co and therefore shifts the magnetic compensation point of the material system. It also has been shown that this finding can be used to design magnetic patterns consisting of areas where the Co magnetic subsystem dominates (Co^+^) the effective magnetization with exception of areas where the Tb subsystem dominates (Tb^+^). With a proper choice of sublayer thicknesses and IB parameters, the changes induced in the two magnetic subsystems preserve perpendicular magnetic anisotropy (PMA) of the whole system. Moreover, these parameters can be chosen so that the Co^+^ areas have lower switching field than the Tb^+^ areas (*H*_S_^Co+^  < *H*_S_^Tb+^). In the hysteresis loops of such layer systems, starting from saturation, it is possible to reverse the Co^+^ areas *only*, so that their effective magnetization is opposite to that of the Tb^+^ matrix. A unique feature of the domains present here is that their boundaries, which coincide with the boundaries between Co^+^ and Tb^+^ areas, contain no DWs. This magnetic pattern is energetically very stable since it minimizes the magnetostatic energy (due to the formation of domains) without a corresponding increase in anisotropy and exchange energy (see also Hrabec et al.^20^). In contrast, when the effective magnetization direction of the layer system does not show lateral variations, we will refer to this state as having no domains, although DWs in the two magnetic subsystems are present (we thus disregard the magnitude of magnetization). Summing up, two peculiar magnetization configurations can be obtained in these systems: effective magnetization domains without DW and a monodomain state (effective magnetization pointing in the same direction everywhere) with DWs in the magnetic subsystems. The influence of these peculiar magnetic configurations on the magnetization reversal and the way the magnetic states of neighboring areas influence the magnetization reversals of the individual domains is not yet fully understood.

To answer these questions, we will first discuss the results of measurements of Ti-4 nm/Au-30 nm/(Tb wedge-0–2 nm/Co-0.66 nm)_6_/Au-5 nm multilayer (sample A) and determine the influence of IB on the properties of that system for various *t*_Tb_ [or equivalently, average Tb concentration (*c*_Tb_)]. Then, we will discuss the results of detailed measurements of the magnetization reversal of a Ti-4 nm/Au-30 nm/(Tb-1.1 nm/Co-0.66 nm)_6_/Au-5 nm multilayer (sample B1 and B2) patterned by IB with two different doses (*D*) D* = 1 × 10^15^ He^+^/cm^2^ and D** = 3 × 10^15^ He^+^/cm^2^. As will become clear below, the D* and D** values were chosen in such a way that for D* the regions modified by IB [two-dimensional (2D) lattice of squares] and protected against IB (matrix) present domination of the Tb subsystem and differ only in their *H*_C_ values. In contrast, for D** the IB modified regions (squares) are Co^+^ with *H*_C_ lower than in the protected Tb^+^ matrix. Note that we have conducted magnetic measurements at room temperature. For both doses, two-dimensional lattices with four different sizes of modified squares were created. A comparison of the behavior of magnetically patterned ferrimagnetic films characterized by the same and different domination of subsystems in modified and protected areas (i.e., patterned with D* and D**, respectively) as well as different size of modified areas allows to determine the role of different energetic contributions to the magnetization reversal. Henceforth, we will refer to the lattices patterned by IB with the two above He^+^ ion doses as D* and D** lattices, respectively. The paper focuses on comparing systems modified with different values of *D* and addresses in sequence the following issues: major loop reversal, squares’ reversal, matrix’s reversal, and minor loop reversal. The reversal processes are studied using hysteresis loops and microscopic observations of magnetic configuration evolution for systems modified with two different doses but the same square size (25 × 25μm^2^).

## Results and discussion

### Modifications of the magnetic properties of Tb/Co multilayers by ion bombardment

**Figure 1:**
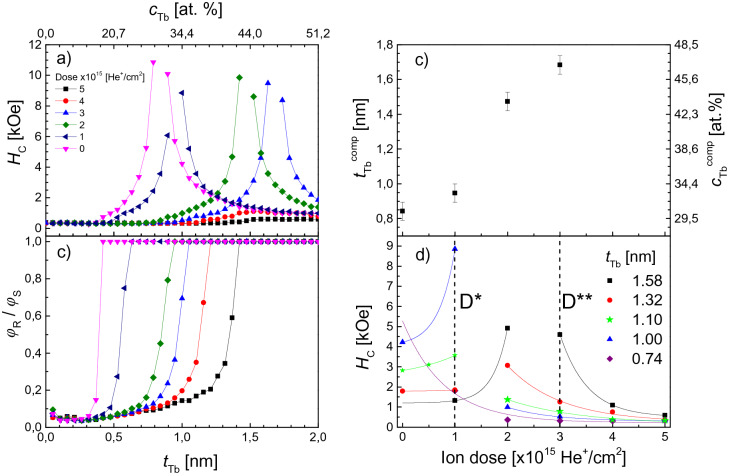
(**a**) *H*_C_ dependence on Tb sublayer thickness (*t*_Tb_) and on average Tb concentration (*c*_Tb_) for the wedged sample A for five different doses, *D*, of 10 keV He^+^ ions, (**b**) ratio between remanence and saturation Kerr signals (*φ*_R_/*φ*_S_) versus *t*_Tb_ (*c*_Tb_), (**c**) Tb sublayer thickness and Tb concentration at the compensation point *t*_Tb_^comp^ and *c*_Tb_^comp^ as a function of *D*, (**d**) *H*_C_(*D*) for various *t*_Tb_ values. D* = 1 × 10^15^ He^+^/cm^2^ and D** = 3 × 10^15^He^+^/cm^2^ indicate doses used in the ion beam patterning experiment described further below.

Measurements results for sample A are shown in Fig. 1. Figure 1a, b show the coercive field *H*_C_ and squareness of the hysteresis loops *φ*_R_/*φ*_S_ as a function of Tb thickness (*φ*_R_ and *φ*_S_ are magnetooptical Kerr signals at remanence and saturation, respectively) for various IB doses in the 0 ≤ *D* ≤ 5 × 10^15^ He^+^/cm^2^ range (*D* = 0 corresponds to the as-deposited system). These results show that for *D* below 3 × 10^15^ He^+^/cm^2^, the compensation thickness and compensation concentration (*t*_Tb_^comp^ and *c*_Tb_^comp^) increase with *D* (Fig. 1c). It is worth noting that before reaching *t*_Tb_^comp^ or *c*_Tb_^comp^, *H*_C_ increases as* D* increases, while for larger *D* a decrease is observed (Fig. 1d). For the two highest doses (*D* = 4 × 10^15^ He^+^/cm^2^ and *D* = 5 × 10^15^ He^+^/cm^2^) the Tb/Co multilayer shows no compensation point at RT in the investigated *t*_Tb_ (*c*_Tb_) range (Fig. 1a). With increasing *D*, the PMA sets in for thicker Tb layers (Fig. 1b), indicating extraordinarily strong magnetic deactivation of Tb. As already discussed^15,43^, this is caused by a preferential oxidation of Tb, relative to Co, resulting in its stronger magnetic deactivation. A possible explanation is that IB creates diffusion paths (vacancy-type defects) that facilitate oxygen diffusion deep into the multilayer structure.

### Magnetization reversal of reference areas

**Figure 2:**
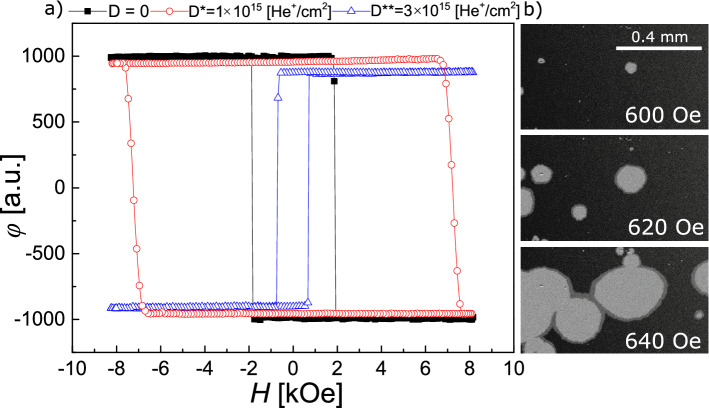
(**a**) Hysteresis loops of the reference areas in the as-deposited state (black squares), after IB with D* = 1 × 10^15^He^+^/cm^2^ (red dots) and D** = 3 × 10^15^He^+^/cm^2^ (blue triangles). (**b**) Representative images of the domain structure of the reference area subject to IB with D**.

Before describing the magnetization reversal of the D* and D** lattices, we analyze the reversal of the reference regions. The corresponding 1 × 1mm^2^ area is large enough so that the magnetization reversal in its center is not influenced by the adjacent areas. The rectangular shape of the hysteresis loops (Fig. 2a) indicates that the reversal of sample B1, as-deposited and after IB with D* and D**, consists of nucleation and fast expansion of a reversed domain^20,44,45^. This statement is confirmed by representative pictures of magnetic structure evolution recorded during magnetization reversal (Fig. 2b). Therefore, the value of the coercive field *H*_C_ is determined primarily by the nucleation process^46^. It can also be seen that sample B1 in the as-deposited state and after IB with D* is Tb^+^, but has larger *H*_C_ in the latter case. In contrast, for IB with D** the modified area is Co ^+^ with lower *H*_C_.

Figure 2b shows the evolution of the magnetic configuration within one of the reference areas (here the one bombarded by D**) for an increasing magnetic field (*H*). Low density of nucleation centers seen in the images (a few of them per mm^2^) and the isotropic propagation of DWs provide evidence for the lateral homogeneity of the layers and the small defect density, not only in protected areas, but in bombarded regions as well. Similar results have been obtained also for all other reference areas in the as deposited samples as well as in those bombarded with different doses.

### Magnetization reversal of 2D lattice patterned with different doses of 10 keV He^+^ ions

#### Major loops: General description

**Figure 3:**
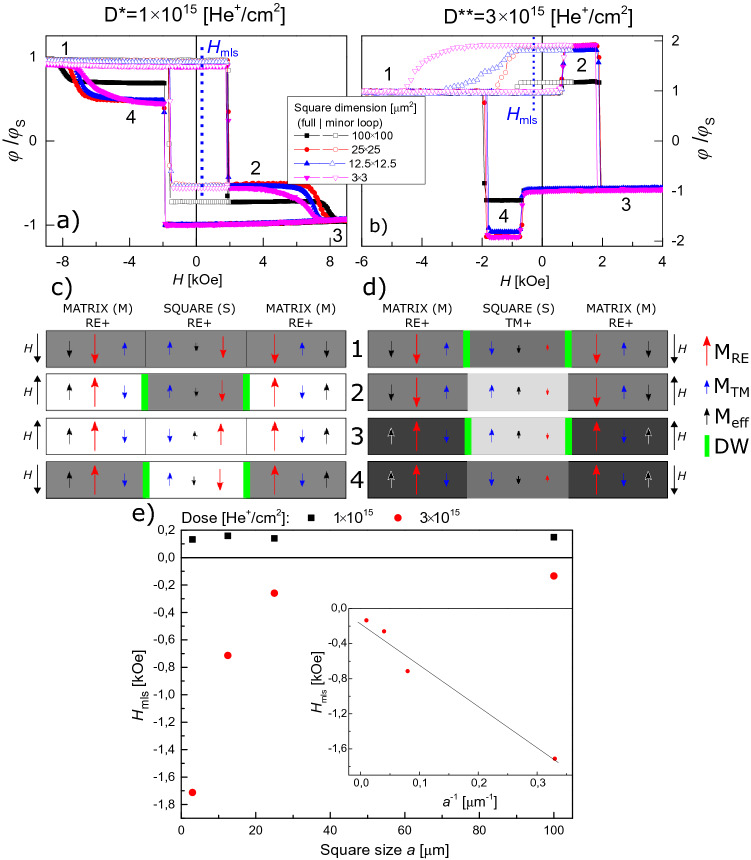
Full and minor hysteresis loops of sample B1, obtained using a Kerr magnetometry in polar configuration, for a 2D lattice of squares with different sizes and patterned with two different doses, (**a**) D* = 1 × 10^15^He^+^/cm^2^ and (**b**) D** = 3 × 10^15^He^+^/cm^2^, *H*_mls_ – indicates the shift of the minor hysteresis loop for a lattice with square sizes of 25 × 25μm^2^. Magnetization orientations of the matrix and the squares for ion beam patterning with D* (**c**) and D** (**d**). The black, blue and red arrows correspond to effective magnetization, magnetization of Co and of Tb subsystems, respectively. The shades of gray correspond to the differential images presented in Figs. 4–9. (**e**) Minor loop shift as a function of square size [*H*_mls_(a)], inset *H*_mls_(1/a).

Figure 3a, b show major and minor hysteresis loops for D* and D** lattices, respectively. In the analysis of the magnetization reversal process recorded for sample B1 with magneto-optical Kerr effect in polar configuration (P-MOKE), it should be taken into account that with the applied light wavelength (*λ* = 655 nm), the signal originates mainly from the Co subsystem^47^. This is clearly reflected in a reversal of the hysteresis loop for Co^+^ regions with respect to Tb^+^ regions. Since the total area of squares (IB modified regions) is three times smaller than that of the matrix (IB protected region) we can clearly recognize the reversal of either squares or matrix in the hysteresis loop. Based on this, Fig. 3c,d contains sketches of magnetic orientations for matrix and squares at the four distinctive stages numbered on the full hysteresis loops (Fig. 3a, b).

**Figure 4:**
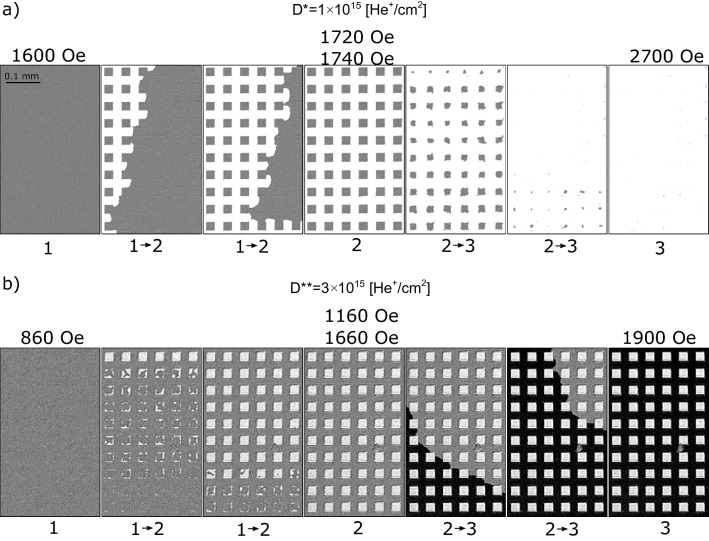
Representative P-MOKE microscopy differential images illustrating the magnetization evolution for the lattice of squares (sample B2, *a* = 25 μm) patterned with ion doses D* and D** (panels (**a**) and (**b**), respectively) during reversal from saturation in negative magnetic field (state 1 in Fig. 3) to saturation in positive field (state 3 in Fig. 3).

For the D* lattice, both squares and matrix are Tb^+^, therefore the orientation of effective magnetization is determined by the magnetization of the Tb subsystem. For loops starting from negative saturation field in this system, the creation of the multi-domain state takes place by magnetization reversal of the matrix since it has a lower value of the switching field (Fig. 3a, c), (see also Fig. 2a). During this process, indicated in Fig. 3a as 1 → 2 transition, DWs are created on the interface between squares and the matrix (Fig. 3c). The corresponding transformation in magnetic structure for the lattice of squares with side *a* = 25 μm (sample B2) recorded using a Kerr microscope is demonstrated in the differential images of Fig. 4a as 1 → 2 transition, where reversed domains grow from nucleation centers located outside of the imaged area. This suggests that the IB does not generate additional nucleation centers within the matrix, which is covered by a thick resist to protect the material underneath from the 10 keV He^+^ ions.

Differential images are, pixel-by-pixel, gray value subtraction of the image obtained in state (1) from the images at a given magnetic field. Since most of the magnetic contrast is due to the magnetization of the Co subsystem, only two shades of gray are visible (Figs. 3c, 4a) for samples with the same orientation of the Co subsystem in squares and matrix in state 1.

The second transition, 2 → 3, corresponds to the reversal of the squares (Fig. 4a). During this process, evolution towards the mono-domain state is accompanied by annihilation of DWs (Fig. 3c). Summing up, the magnetization reversal of a thin film ferrimagnetic heterostructures composed of squares embedded in a matrix with the same subsystem domination (Tb^+^ in this case) is very similar to that of more common magnetically patterned ferromagnetic structures^45,48^. One crucial difference between these systems is that the saturation magnetization is much higher for ferromagnetic films than for near-compensation ferrimagnetic films. Therefore, for the same film thicknesses, shape and element sizes, the magnetostatic interactions will be weaker in 2D ferrimagnetic lattices making them attractive for memory applications. The weak interactions between individual squares and between the squares and the matrix in a ferrimagnetic system is confirmed by the similarity of *H*_C_ values of reference regions (black and red curve in Fig. 2a) and the switching fields, *H*_S_, visible in the major loop of Fig. 3a.

The shape of the hysteresis loop measured for the D** lattice and presented in Fig. 3b differs from those presented in Fig. 3a. Although the magnetic properties of the matrix (IB protected area) are the same (Tb^+^) the increase of dose from D* to D** results in more pronounced weakening of the Tb subsystem and, in consequence, a transition of the squares to Co subsystem domination (Co^+^).

In the differential images of Fig. 4b, the varying effective magnetization produces three distinct shades of gray. This situation is different from the one presented in Fig. 4a because now the Co subsystem magnetization orientation in the squares and in the matrix are antiparallel to each other for state 1. The magnetization directions of the Co and the Tb subsystems shown in Fig. 3d were inferred following the magnetization reversal presented in Fig. 3b. States 1 and 3 (Fig. 3b) occur at saturating fields where the effective magnetization points in the same direction in the whole lattice. Even though this is a monodomain state with respect to the direction of the effective magnetization, there are DWs within the magnetic Co and Tb subsystems at the interfaces between the Co^+^ squares and the Tb^+^ matrix. Here the moments of the Co and Tb atoms maintain their antiparallel orientation by a coupled rotation^16^. On the other hand, in states 2 and 4, i.e., after reversal of squares (*H*_S_^squares^ < *H*_S_^matrix^) (Fig. 3b, d), the effective magnetizations inside the squares and in the matrix have opposite directions, but there are no DWs within the two magnetic subsystems on the borders of the squares. Due to this unique situation, states 2 and 4 are energetically favorable because they lower the magnetostatic energy by forming domains, without a corresponding increase in anisotropy and exchange energies associated with the formation of DWs.

#### Reversal of squares

**Figure 5:**
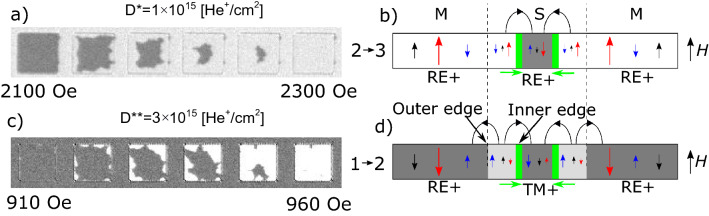
(**a**, **c**) Close-up image of one representative square with *a* = 25 µm illustrating the magnetization reversal for 2 → 3 and 1 → 2 transitions after IB with dose D* and D**, respectively. (**b**, **d**) Sketch of the effective magnetization, and the magnetization of the Co and Tb subsystems (with the color convention of Fig. 3) in cross sectional view. The stray fields from magnetic domain structure are schematically presented in (**b**) and (**d**). Green arrows show the direction of DWs movement.

For each individual square, under the influence of the external positive field (*H* > 0), the systems undergo 2 → 3 and 1 → 2 transitions for D* (Fig. 5a) and D** (Fig. 5b) lattices, respectively, by an inward motion of the DWs. In both systems the walls at the boundaries between squares and matrix already exist and the reversal takes place by their inward movement. Notwithstanding this similarity, both cases show clear differences. For squares with the same subsystem domination as in the matrix (Fig. 5a, b) the reversed region of squares (region between square edge and DW) has the same orientation of the effective magnetization as the matrix. In contrast, for the system presented in Fig. 5c, d, the inward motion of the DW leaves behind a new effective magnetization domain close to the edges of the squares. The effective magnetization of this domain is antiparallel to that of the matrix, and also to that at the square’s center because the center has not yet reversed (Fig. 5d). Therefore, the stray fields acting on the reversed region are higher for the system presented in Fig. 5c, d. In our opinion, this difference explains why the field range for reversals of the D** lattice is narrower than that of the D* lattice (Figs. 3, 4, 5).

#### Reversal of matrix

**Figure 6:**
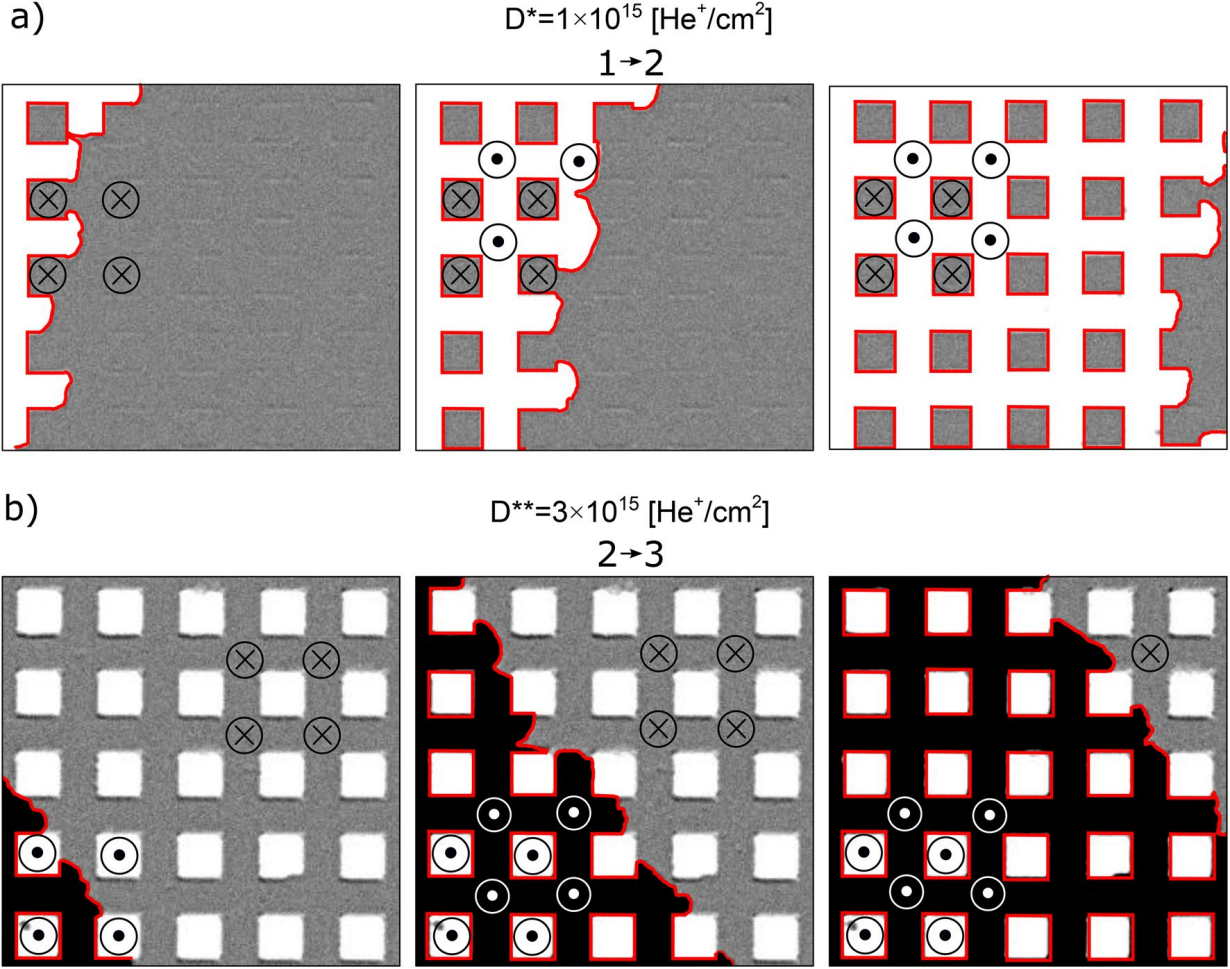
Snapshots of DW propagation during the matrix reversal for the system with *a* = 25 μm (sample B2) magnetically patterned with two different ion doses.

For D* and D** lattices, the magnetization reversal of the matrix (processes 1 → 2 and 2 → 3) takes place by DW propagation from few nucleation centers (Fig. 4, 6). However, during the 1 → 2 transition (Fig. 6a) domains and DWs are created and during the 2 → 3 transition (Fig. 6b) domains are annihilated together with the formation of DWs. As the DW expands through the lattice, it extends and wraps around the perimeter of each square as shown by the red lines in Fig. 6. DW propagation is slightly delayed when crossing the squares. After the matrix reversal, the final state for the D* lattice is a multi-domain structure with DWs (state 2); and for the D** lattice (state 3), a monodomain state with DWs (Fig. 6).

### Minor loop: Magnetization reversal

**Figure 7:**
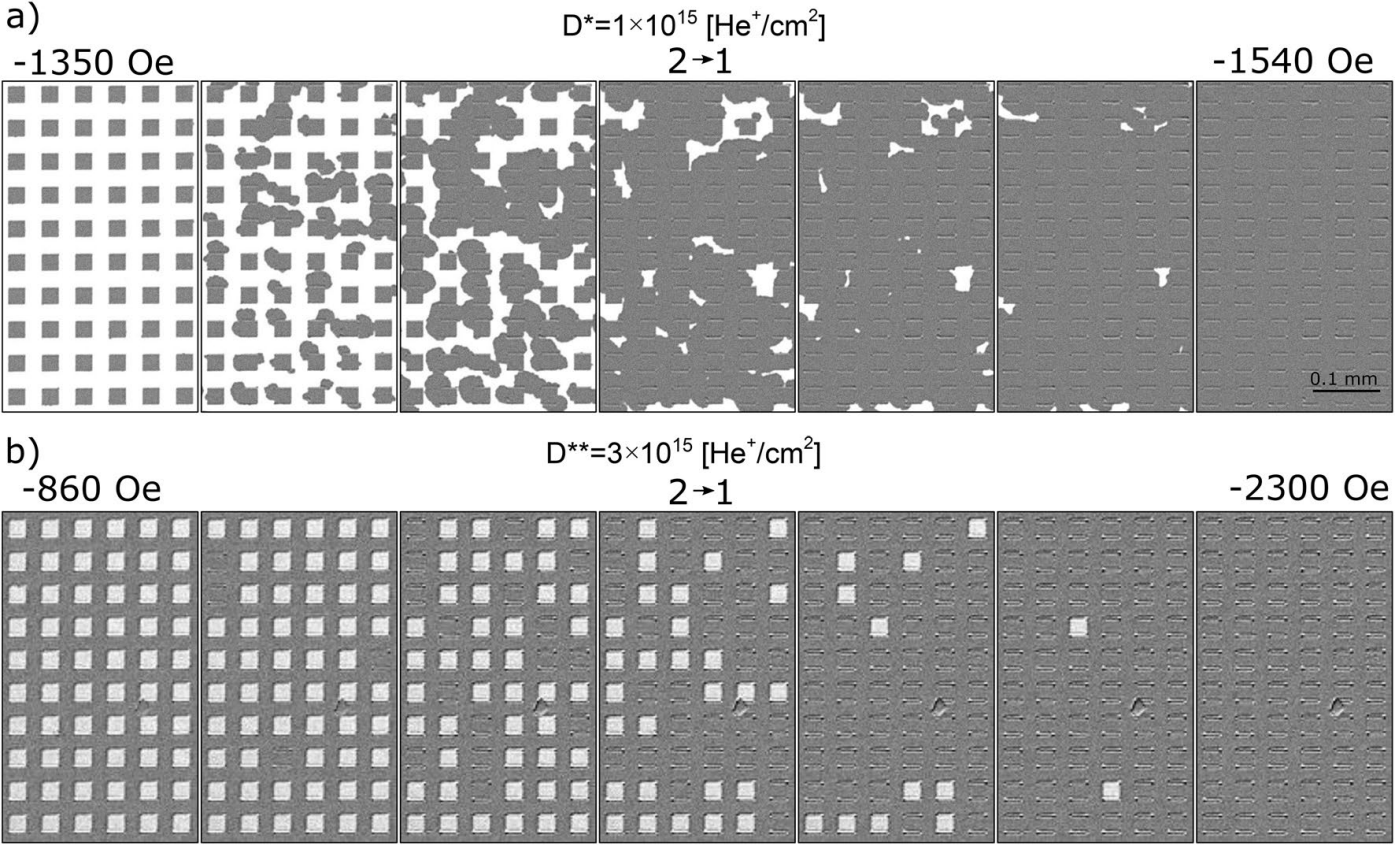
Magnetization reversal of squares in the lattice with *a* = 25 μm (sample B2), corresponding to the transition 2 → 1 for lattice patterned with (**a**) D* = 1 × 10^15^He^+^/cm^2^ and (**b**) D** = 3 × 10^15^He^+^/cm^2^.

Minor loop measurements (Fig. 3a, b) and magnetic domain structure images (Fig. 7) provide information about the interaction between the IB modified squares and the protected matrix.

For the D* lattice the minor loop reflects reversal of the matrix (Figs. 4a, 7a); and for the D** lattice, reversal of the squares (Figs. 4b, 7b). The transition 1 → 2 for both lattices was described in the discussion of the full hysteresis loops. The backward transitions, i.e., 2 → 1, is not just a reversal of the 1 → 2 transition. In the case of the D* lattice in state 2 (Figs. 3c, 7a) the multidomain state already exists, therefore the matrix’s reversal starts by DWs propagation without nucleation. Consequently, the switching field during transition 1 → 2 is higher than that associated with the 2 → 1 transition (*H*_S_^12^ > *H*_S_^21^). Consistent with this, the field value corresponding to the minor loop shift (*H*_mls_) is positive (Fig. 3a). This interpretation of small asymmetries of minor loops requires that *H*_mls_ is insensitive to the sizes of the squares (Fig. 3e).

For the D** lattice in state 2, the effective magnetizations of squares and matrix are antiparallel to each other (multi-domain state), but on the interfaces between them there is no magnetization rotation of Co and Tb subsystems (DWs are not present). As mentioned before and in previous papers^15,19^ this unique magnetic configuration is energetically very stable because it lacks DWs between domains and the reduction of demagnetizating energy is accompanied by a decrease of anisotropy and exchange energy. Thus, the transition 2 → 1 takes place by annihilation of domains and creation of DWs. Therefore, it implies higher energy cost than the 1 → 2 transition. This interpretation is confirmed by the relation *H*_S_^12^ < *H*_S_^21^, which is opposite of what was determined for the lattice patterned with D* and because *H*_mls_ is now negative (Fig. 3b). Moreover, |*H*_mls_| decreases with *a* (Fig. 3e). These tendencies confirm our proposed interpretation since the energy term related to DW creation in magnetic films is proportional to the total DW length, which is inversely proportional to *a*.

The demagnetizing energy depends mainly on the saturation magnetization, and the DW energy on the exchange and anisotropy constants. The variability of these parameters influences the annihilation of domains and creation of DWs during 2 → 1 transition. These variations are, in our opinion, responsible for the relatively large lateral distribution of *H*_S_^21^ values. One may further speculate that the partial magnetic deactivation of the Tb subsystem contributes to significant local variations of all the mentioned parameters.

**Figure 8:**
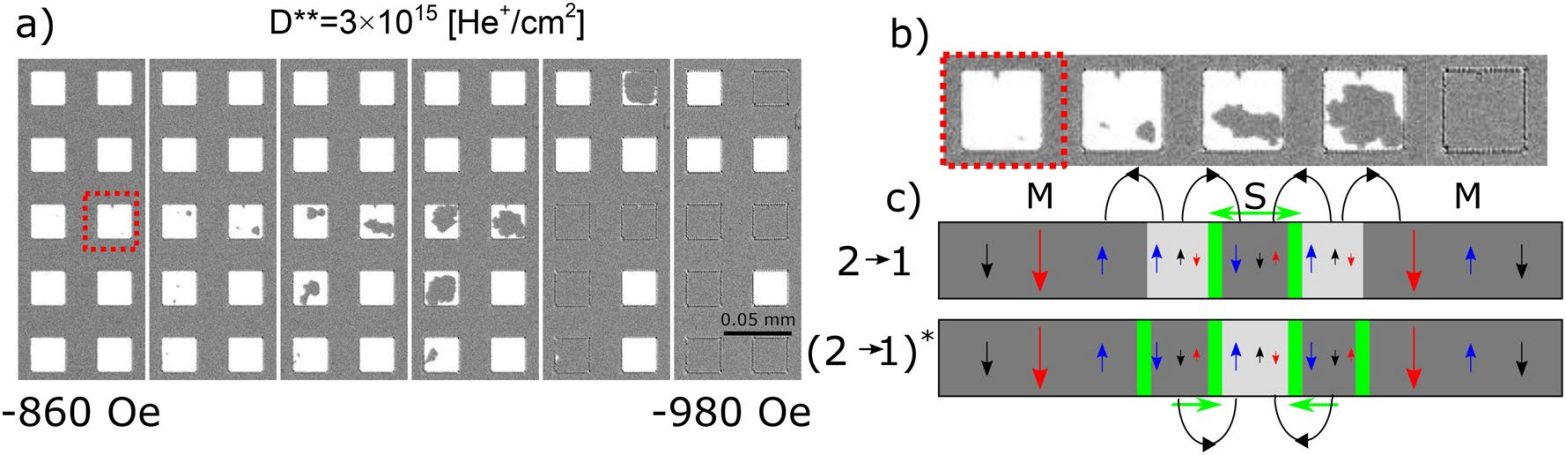
(**a**) P-MOKE microscopy images of the magnetization reversal of squares with *a* = 25 μm (sample B2) for the 2 → 1 transition and (**b**) close-up image of one representative square. (**c**) Schematic cross-section of the intermediate phase showing the effective magnetization, and the magnetization of the Co and Tb subsystems within the matrix (M) and the square (S) in two variants: (2 → 1) with the domain nucleation in the center of the square [consistent with the images of panel (a)], (2 → 1)* with the domain nucleation in the vicinity of the edge of the square (this requires two DWs: one at the edge of the square and another close to its center). The stray fields from magnetic domain structure are schematically presented (**c**).

Figure 8a shows that the transition 2 → 1 for the D** modified system begins with a nucleation near the center of the square, which is the opposite of the 1 → 2 transition (Fig. 5b). To explain the origin of the difference between the 1 → 2 and the 2 → 1 transitions we recall that state 1 contains DWs and therefore the squares’ 1 → 2 reversal occurs by propagation of these DWs (Fig. 5c, d). In contrast, state 2 contains no DWs and the process starts by nucleation of a reversed domain. The nucleation takes place in the central part of squares. To explain why, we show a schematic cross-section of two potential transition pathways in Fig. 8c, labeled (2 → 1) and (2 → 1)*. The difference between these two potential pathways is the location in the square where the nucleation sets in. In the first scenario [labeled (2 → 1)], the nucleation occurs near the square’s center, consistent with the images shown in Fig. 8a, b. In the second possible scenario, [labeled (2 → 1)*], the domain nucleation occurs at the square’s edge (where *H*_C_ is low). The second scenario requires the creation of two DWs, one on the inner edge and the other in the outer edge of the nucleated domain. The (2 → 1)* transition requires thus more energy than the (2 → 1) transition so it is less likely to occur.

The intra-square reversal process just described could be registered only for squares with low nucleation field, i.e., for squares characterized by small differences in energy associated with nucleation of reversed domains and DW propagation. In contrast, for squares with high nucleation fields the magnetization reversal takes place abruptly even for slow field-sweeps, thus the DWs displacement could not be observed (Fig. 7b).

### Minor loop after partial reversal of the matrix

**Figure 9:**
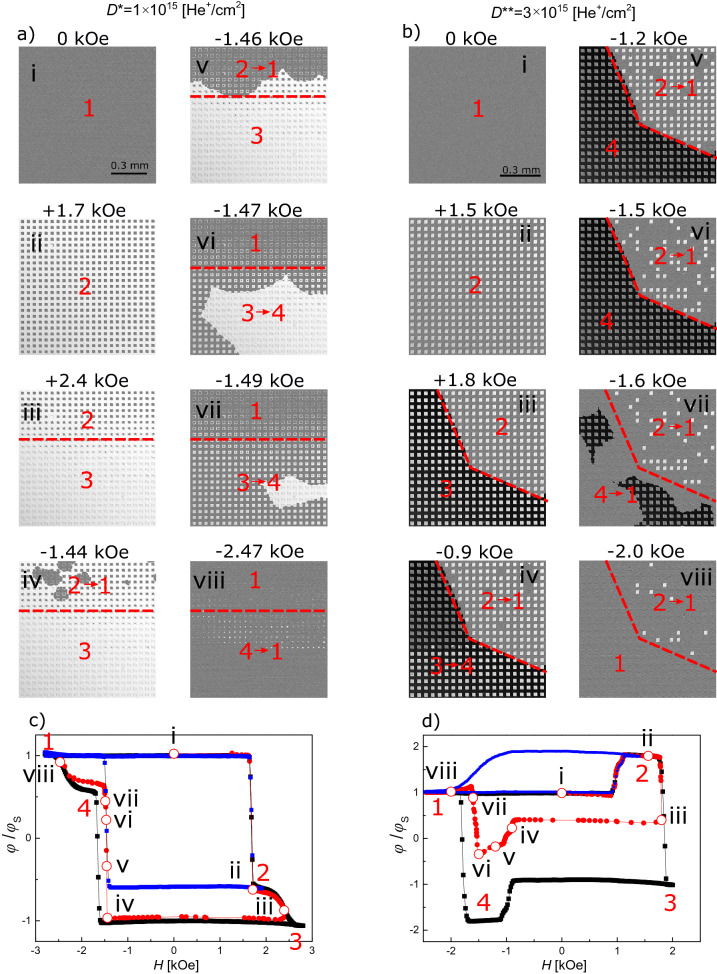
(**a**, **b**) Representative differential images illustrating the magnetization evolution in lattices of squares with *a* = 25 μm (sample B2) during minor loop reversal starting from saturation in negative magnetic field. The maximum positive field was chosen to induce a partial reversal of the magnetically hard areas (i.e., either the squares for D* lattices or the matrix for D** lattices). (**c**, **d**) Hysteresis loops obtained from gray values of the images of IB modified lattices (major loop in black, minor in blue, and red for the loop shown in the two top panels). The left column of the figure (**a**, **c**) corresponds to data obtained from D* lattices; the right side (**b**, **d**), to data from D**.

In previous parts of the work we showed that, in ferrimagnetic planar heterostructures, the magnetization reversal of ion modified squares embedded in an unbombarded matrix requires smaller fields when DWs exist at the boundary between squares and matrix. In other words, the reversal processes based on the annihilation of DWs is easier than the processes that require their creation. It should be noted that it is irrelevant whether DWs separates areas of the opposite or the same effective magnetization orientation. To corroborate this hypothesis, we have performed, for both lattices (D* and D**), studies of the magnetization reversal with partially reversed magnetically harder areas, i.e. squares and matrix, for the D* and D** lattices, respectively (Fig. 9). For these processes, Fig. 9a, b shows how the magnetization evolves. In Fig. 9c, d the corresponding hysteresis loops (red symbols) are compared with major loops (black symbols) and minor loops (blue symbols) measured with maximum positive field enabling reversal of only the softer part of the lattice. The magnetization configurations for the black and blue hysteresis loops shown in Fig. 9c, d are presented in Fig. 4 and in Fig. 7, respectively. All hysteresis loops presented in Fig. 9c, d were determined from microscopic observations of changes to the domain structures during magnetic field variation.

To facilitate the analysis of the magnetization configuration, numbers corresponding to the states defined in Fig. 3c, d are shown on each panel of Fig. 9a, b. The first two panels (i) and (ii), for both D* and D** lattices, correspond to states 1 and 2, respectively. Panels (iii) show the magnetization configuration for the maximum applied positive field, at which the squares and matrix, correspondingly, were partially reversed. For these panels, the red dashed line separates the area in which the squares in the D* lattice and the matrix in D** lattice have not been reversed (i.e. they are in state 2) from the area in which they have been reversed (they are in state 3). It is worth noticing that DWs occur in state 2 for the D* lattice and in state 3 for the D** lattice. After changing the magnetic field to negative values (panels iv-viii), the magnetization configuration evolves in separate regions of the sample following two possible paths. These two reversal paths are analogous to either a major loop process (transition 1 → 2 → 3 → 4) or a minor loop process (transition 1 → 2 → 1). The major loop path occurs in the state 3 of panels (iii) and the minor loop path in state 2 areas. For the D* lattice, the existence of DWs in state 2 triggers the matrix reversal in this area (Fig. 9a panels (iv) and (v)). The DWs propagating in the matrix reach the border between the state 2 area and the state 3 area (indicated by the red line) and, as the propagation continues, the matrix undergoes the 3 → 4 transition (Fig. 9a panels (vi) and (vii)). Therefore, the magnetic field values triggering the matrix reversal in transitions 2 → 1 and 3 → 4 are similar. In contrast, since the D** lattice supports domains without DWs (state 2) and DWs without domains (state 3), the field difference is very large for the squares’ reversal that occurs during transitions 2 → 1 and 3 → 4 (Fig. 9b panels (iv)-(viii)). This is clearly visible in Fig. 9b panel (v) where the reversal of all squares below the red line (state 4) is achieved at |*H*|≈1.2 kOe whereas almost all squares, above the red line, remain in state 2. To switch a substantial number of squares above the red line, a large increase of magnetic field (|*H*| > 2 kOe), is required. This will force the transitions 2 → 1 and corroborates our previous statements about the stability of the magnetization configuration that contains domains without DWs.

## Conclusions

In a rare earth–transition metal ferrimagnetic system He^+^ ion bombardment shifts the compensation point of the two magnetic subsystems towards a higher concentration of rare-earth. This effect can be used for local magnetic patterning and to fabricate a 2D-lattice of artificial magnetic domains. For a prototype Tb/Co system that, as-deposited, is Tb dominated (Tb^+^), we have shown how to adjust the applied ion dose to create areas that are still dominated by Tb or, for higher dose, by Co (Co^+^). We have demonstrated that the lattice consisting of Tb^+^ squares embedded in the Tb^+^ matrix behaves as the more common IB patterned ferromagnetic films. Conversely, within lattices in which Co^+^ squares are embedded in a Tb^+^ matrix, we create effective magnetization domains without domain walls in either magnetic subsystem. These domains are particularly stable because they show a deep minimum in their free energy due to flux closure of the stray fields and corresponding energy reduction without exchange and anisotropy energy increases associated with domain walls. In contrast, in magnetic saturation the corresponding monodomain state of the effective magnetization shows domain walls in the two magnetic subsystems. For both lattices, this work provides a comparative analysis of magnetization reversal data obtained using a magnetooptical magnetometry and microscopy. This analysis clearly shows that for both types of lattices the magnetization reversal takes place by the propagation of DWs and, if they are not present, the reversal starts from nucleation of reversed domains followed by propagation. It is not important whether a DW separates areas with the same or with opposite orientation of the effective magnetization.

## Experimental/method

Experiments have been carried out on three samples: (A) Si/Ti-4 nm/Au-30 nm/(Tb-wedge 0–2 nm/Co-0.66 nm)_6_/Au-5 nm, and (B1, B2) Si/Ti-4 nm/Au-30 nm/(Tb-1.1 nm/Co-0.66 nm)_6_/Au-5 nm. The deposition process and the IB procedure have been described elsewhere^15^.

(A) type sample has been fabricated to investigate the influence of He^+^ 10 keV IB on the magnetic properties of this material system, particularly on the coercivity and on the compensating Tb concentration *c*_Tb_. Magnetic measurements have been performed using P-MOKE magnetometry for an ion dose in 1 × 10^15^ He^+^/cm^2^ ≤ *D* ≤ 5 × 10^15^ He^+^/cm^2^ range, extending the one of the preceding investigations^15^. The P-MOKE characterization has been performed using a 655 nm wavelength laser with a spot diameter of about 0.3 mm. Photo-elastic modulation and phase-sensitive detection were used to increase the signal-to-noise ratio.

Magnetic properties of magnetically patterned Tb/Co layers were determined for sample (B1) with a P-MOKE magnetometry and (B2) with a P-MOKE microscope. For sample (B1) patterns consisting of lattices of squares with side lengths *a*, and distances *2a* between neighboring squares’ centers (*a* = 3, 12.5, 25, and 100 μm) have been fabricated by a combination of photolithography and IB with D* = 1 × 10^15^ He^+^/cm^2^ and D** = 3 × 10^15^ He^+^/cm^2^. The total area of each lattice was 1 × 1 mm^2^. In addition, a 1 × 1 mm^2^ area has been uniformly bombarded (D* = 1 × 10^15^ He^+^/cm^2^ and D** = 3 × 10^15^ He^+^/cm^2^) as a reference. For sample (B2) patterns consisted only of squares with *a* = 25 μm.

Note that experiments were performed using two separate samples, B1 and B2, with the same morphology and parameters of IB modification. Thus, samples B1 and B2 have similar magnetic properties; however, their switching fields are different. This difference between switching fields is strikingly large for the transition 2 → 3 (compare Figs. 3a, b and 9c, d). This is because, near compensation, very small changes in Tb/Co composition cause large *H*_S_^42^ variations.

The P-MOKE apparatus has been a Zeiss Microscope adapted by Evico Magnetics GmbH, Dresden. Movies were recorded for continuous field sweeps with a frame rate between 10 and 20 fps. To improve the image quality, we used zero-field recording technique (remanence state images were taken with frozen magnetic structure after applying given magnetic fields). To further improve contrast, we subtracted the image obtained at given field from the reference image taken at negative saturation. All magnetic measurements were performed at room temperature with a magnetic field perpendicular to the sample plane.

## Data Availability

The data of this study are available from the corresponding authors on reasonable request.
